# Colorectal Cancer: Genetic Underpinning and Molecular Therapeutics for Precision Medicine

**DOI:** 10.3390/genes15050538

**Published:** 2024-04-25

**Authors:** Gideon T. Dosunmu, Ardaman Shergill

**Affiliations:** Section of Hematology/Oncology, Department of Medicine, University of Chicago, Chicago, IL 60637, USA; gideon.dosunmu@uchicagomedicine.org

**Keywords:** colorectal cancer, genetic alterations, precision medicine, molecular therapeutics

## Abstract

Colorectal cancer (CRC) accounts for about 10% of all cancer cases and 9% of cancer-related deaths globally. In the United States alone, CRC represents approximately 12.6% of all cancer cases, with a mortality rate of about 8%. CRC is now the first leading cause of cancer death in men younger than age 50 and second in women younger than age 50. This review delves into the genetic landscape of CRC, highlighting key mutations and their implications in disease progression and treatment. We provide an overview of the current and emerging therapeutic strategies tailored to individual genomic profiles.

## 1. Introduction

Colorectal cancer (CRC) is a major health challenge globally. It is the third most common cancer worldwide, with approximately 1.8 million new cases and over 860,000 deaths annually [1]. This accounts for about 10% of all cancer cases and 9% of cancer-related deaths globally. In the United States alone, CRC represents approximately 12.6% of all cancer cases, with a mortality rate of about 8% [2]. CRC is now the first leading cause of cancer death in men younger than age 50 and second in women younger than age 50 (after breast cancer) [3]. Colon and rectal cancers are a set of complex diseases marked by various genetic alterations and molecular mechanisms [4]. This review delves into the genetic landscape of CRC, highlighting key mutations and their implications in disease progression and treatment. We provide an overview of the current and emerging therapeutic strategies tailored to individual genetic profiles of metastatic CRC (mCRC).

## 2. Search Strategy

Our search strategy, (Table 1) in the PubMed database involved the utilization of specific MeSH terms, including “Colorectal Neoplasms”, “Metastatic “, “Molecular Targeted Therapy”, “Precision Medicine”, and “Mutation”, along with related terms such as “BRAF “, “KRAS “, “NRAS “, “ERBB2 “, “Microsatellite Instability”, “DNA Mismatch Repair”, “Immunotherapy”, “Antineoplastic Agents “, “Biomarkers, Tumor”, “Circulating Tumor DNA”, “Drug Resistance, Neoplasm”, and “Clinical Trials”. To ensure comprehensiveness, we also examined the references of pertinent articles to capture any studies that may have been overlooked. Additionally, we searched ClinicalTrials.gov for information on current clinical trials. We looked at abstracts from ASCO and ESMO between 2021 and 2024. The focus of the data extraction was on identifying genetic mutations, molecular pathways implicated in colorectal cancer, and the corresponding therapeutic outcomes.

## 3. Molecular Alterations and Prevalence in mCRC

### 3.1. BRAF and RAS Mutations

*BRAF* mutations, particularly the *V600E* variant, are found in about 6–8% of metastatic CRC cases [5]. These mutations are a significant prognostic factor and are mutually exclusive with *KRAS* mutations [6]. They are more common in patients with mismatch repair deficiency (d-MMR) [7] and lead to the activation of the *MAPK* signaling pathway, affecting the serrated pathway of CRC development [8]. Clinically, *BRAF V600E* mutations in metastatic CRC are associated with a lower overall survival (OS) and resistance to traditional chemotherapy and anti-*EGFR* therapy [9].

*Non-V600 BRAF* mutants, which are present in about 2–3% of CRC cases, typically occur in younger patients with left-sided disease and have a more indolent course. They result in a better overall survival compared to *V6 [00]E* mutation carriers, but a worse survival compared to patients with wild-type *BRAF* [10,11].

**Figure 1:**
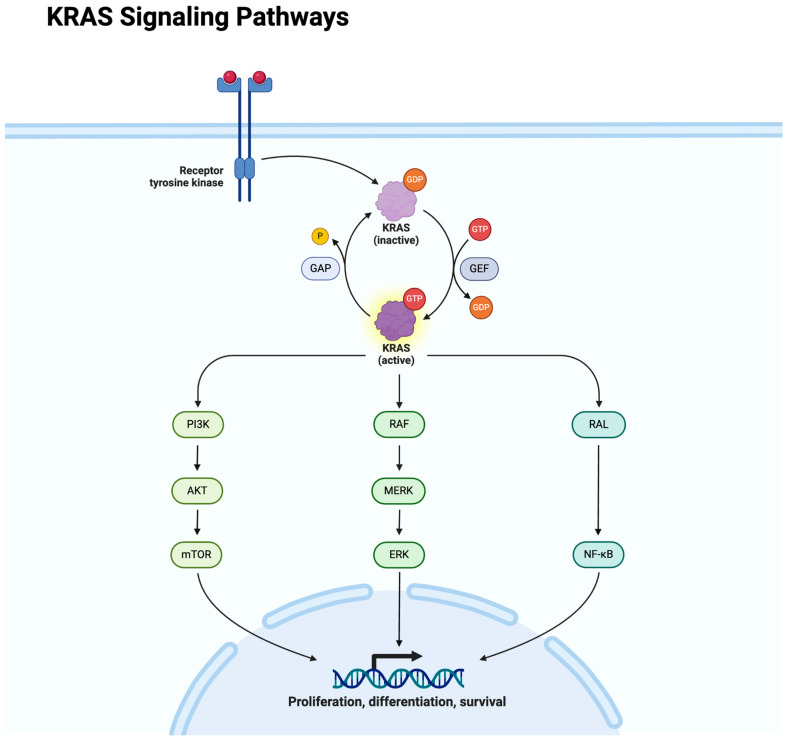
Created with BioRender.com.

A total of 61.3% of mCRC cases are *RAS* wild-type with no mutations, while *KRAS* mutations account for 40 to 45% of cases (Table 2). *NRAS* and *HRAS* mutations are less common, found in 5.2% and 1.0% of cases, respectively [12]. Mutations in *KRAS* and *NRAS* genes at codons 12, 13 (exon 2); 59, 61 (exon 3); and 117, 146 (exon 4) are considered predictors of resistance to *EGFR*-targeted therapies. These mutations do not usually occur together [13]. They activate the *RAS/RAF/MAPK* signaling pathway (Figure 1), leading to uncontrolled cell division and cancer progression [12]. These mutations have significant clinical implications, as they are associated with resistance to anti-*EGFR* therapies such as cetuximab and panitumumab. Patients with *KRAS* or *NRAS* mutations typically do not respond to these treatments and often have poorer outcomes. Conversely, patients with wild-type KRAS or NRAS usually respond better to anti-*EGFR* therapies. In wild-type *KRAS*, anti-*EGFR* therapies retain their ability to bind to and inhibit the EGFR, disrupting the downstream signaling of the *RAS/RAF/MAPK* pathway [14]. Of notable mention are *KRAS* mutation variants due to their lower overall survival and limited treatment options after progression on first-line therapies. The *G12D* variant is the most prevalent, accounting for 13% of *KRAS* mutations. This is followed by the *G12V* variant at 9%, the *G13D* variant at 7.4%, and the *G12C* mutation at 3.2% of cases [15].

### 3.2. HER2 (Human Epidermal Growth Factor Receptor 2) Amplification

*ERBB2* amplification occurs in approximately 2–9% of metastatic cases [16]. This amplification is more frequent in rectal cancer than colon cancer and is associated with HER2 positivity. The *HER2* gene, encoded by *ERBB2*, functions as a receptor tyrosine kinase. Its amplification leads to the activation of replication signals independent of ligand-bound dimerization partners. Clinically, HER2 amplification has been linked to resistance to anti-EGFR therapies in metastatic colorectal cancer [17].

HER2-targeted therapy trials in mCRC are informed by the disease’s molecular biology, notably the role of the tyrosine kinase receptor *HER2* in driving tumor growth when overexpressed. This guides the selection of patients for *HER2*-directed therapies, especially those with *HER2* amplification and without *RAS/BRAF* mutations.

### 3.3. Microsatellite Instability (MSI-H) and Mismatch Repair Deficiency (d-MMR)

Microsatellite instability—high (MSI-H) tumors represent a subset of cancers that are characterized by a deficiency in the DNA mismatch repair (MMR) system. They involve proteins such as MLH1, MSH2, MSH6, and PMS2. Approximately 16% of all CRCs and about 4% of metastatic cases have frameshift mutations in microsatellite DNA [18,19]. The MMR system is responsible for correcting DNA replication errors, and its deficiency leads to a hypermutated state within the tumor genome. This hypermutation increases the number of neoantigens on tumor cells, making them more recognizable to the immune system [20].

Recent advances in cancer immunotherapy have shown that MSI-H tumors are particularly responsive to programmed death 1 (PD-1) inhibitors. PD-1 is a checkpoint protein on T cells that, when activated, dampens the immune response. Tumors can exploit this pathway to escape immune detection. Drugs that inhibit PD-1, such as pembrolizumab and nivolumab, can block this interaction, thereby enabling the immune system to attack cancer cells [21].

### 3.4. NTRK Gene Fusions

Neurotrophic tropomyosin receptor kinase (*NTRK*) gene fusions in colorectal cancer are rare, but clinically significant. They occur in less than 2.5% of colorectal cancer cases. They are predominant in those with right-sided, microsatellite instability—high, and *RAS/BRAF* wild-type tumors. Their underlying mechanism involves the constitutive activation of the *NTRK* kinase domain due to inter- or intra-chromosomal rearrangements [22]. Clinically, identifying patients who may benefit from targeted treatments as a result of *NTRK* gene fusions is important, especially in settings where there are no other effective treatment options. Larotrectinib and entrectinib, which have both been approved as tissue-agnostic drugs for *NTRK*-rearranged tumors by the FDA, have shown promise.

## 4. *FGFR* Alterations and *MET* Amplification

Alterations in fibroblast growth factor receptors (*FGFRs*) occur in approximately 4% of colorectal cancer cases. These receptors are part of the receptor tyrosine kinase family. They are integral to various cellular functions such as growth, survival, migration, and differentiation. In colorectal cancer, changes in certain *FGFRs*—namely *FGFR1*, *FGFR2*, and *FGFR3*—are known to disrupt normal cellular signaling. This disruption typically manifests as increased cellular proliferation, survival, and angiogenesis. These significantly contribute to the advancement and progression of tumors [23]. Targeting these altered FGFRs has become a focal point in the development of new therapeutics. Medications such as pemigatinib and erdafitinib have been formulated specifically to inhibit these receptors. Broader-spectrum drugs such as ponatinib and dovitinib also target FGFRs and other kinases [24].

*MET* amplification is uncommon in untreated cases, but is found in about 20% of patients resistant to anti-*EGFR* monoclonal antibody therapy. Patients with focal MET amplification have a notably shorter overall survival after anti-*EGFR* monoclonal antibody treatment [25]. The *MET* proto-oncogene, found on chromosome 7q31, produces a receptor essential for tumor growth and spread. It is a key target in cancer research, with trials focusing on inhibiting the *MET* pathway [26].

### 4.1. RET Fusion

*RET* mutations and fusions are well established in cancers such as NSCLC and thyroid cancers. Their presence in mCRC is less frequent, yet clinically significant. *RET* fusions in mCRC are reported in approximately less than 1% of cases [27]. Selective *RET* inhibitors such as selpercatinib (LOXO-292) have demonstrated promising results. For instance, selpercatinib showed a 68% ORR in *RET*-fusion-positive NSCLC [28] and a 69% ORR in *RET*-mutant MTC [29]. Extrapolating from these results, targeted therapies against RET fusions could be a promising area for further clinical research in colorectal cancer. Clinical trials are ongoing. The rationale for the potential efficacy of RET inhibitors in colorectal cancer lies in the fact that *RET*, a receptor tyrosine kinase involved in cell growth and differentiation, can become constitutively active through genetic rearrangements such as fusions in some cancers, including a subset of colorectal cancers. This leads to uncontrolled cell proliferation and the survival of tumors. This aberrant *RET* signaling activates various downstream pathways such as *MAPK/ERK*, *PI3K/AKT*, and *PLCγ*, which are critical for cell proliferation and survival [30].

### 4.2. TP53 Mutations

One of the most common somatic gene mutations in colorectal cancer is the *TP53* mutation, which is present in approximately 50% of all CRC cases [31]. It plays an important role in the malignant transformation of adenomas into carcinomas. The TP53 gene, which is found on chromosome 17’s short arm, is a critical tumor suppressor protein that regulates the cellular cycle and apoptosis, acting as the “guardian of the genome”. These mutations occur later in the adenoma–carcinoma progression, driving the development of CRC through the loss of heterozygosity and missense mutations. Through these mutations, growth suppression is deregulated [32]. *TP53* mutations are associated with poor prognoses in CRC patients, with studies indicating a link between these mutations and advanced disease progression, particularly when they occur in combination with other genetic alterations such as KRAS mutations [33]. While research continues into developing new agents targeting pathways affected by *TP53* mutations, these advances are in the early stages and are not yet ready for clinical application.

In the phase 1 study of PC14586, targeting the *TP53 Y220C* mutation in cancer, 29 patients with various advanced solid tumors, including colorectal cancer tumors, were treated. The drug was well tolerated, with most adverse events being mild. Of the 21 patients evaluable for efficacy, five showed partial responses, especially in higher-dose cohorts. Additionally, seven patients achieved a stable disease. The decreases in mutant *p53* circulating DNA and tumor cells suggested effective targeting by *PC14586*. These results indicated preliminary efficacy in heavily treated patients [34].

### 4.3. APC Mutation

Up to 70% of sporadic colorectal cancer cases are caused by *APC* mutations. They play a key role in disease progression and treatment outcomes [35]. *APC* is located on chromosome 5q21-q22. The *APC* gene is important for the regulation of the cell cycle due to its interaction with beta-catenin, which is part of the cadherin adhesion complex and the Wingless/Wnt pathway [36]. These mutations are responsible for familial adenomatous polyposis (FAP), are often found at the initial stages of colorectal neoplasia, and are predominantly associated with the classic tubular adenoma pathway and chromosomal instability (CIN) cancers. As a result of *APC* mutations, beta-catenin’s nuclear translocation and subsequent overactivation of Wnt signaling are disrupted, resulting in excessive cell proliferation and activation. Clinically, these mutations are linked to poorer survival outcomes in advanced stages of CRC [37]. Despite the critical role of WNT pathway activation in CRC pathogenesis, it has been difficult for *APC* mutation knowledge to be directly applied in clinical settings in terms of treatment selection or early cancer detection. The development of targeted inhibitors has remained in preclinical testing.

## 5. Therapeutic Targeted Strategies in mCRC

### 5.1. BRAF Inhibitors

Encorafenib is a *BRAF* inhibitor that is being explored in clinical trials. The BEACON study (Table 3), a phase-3 open-label trial, enrolled 665 patients with *BRAF V600E*–mutated metastatic CRC who had progressed after one or two previous treatments. The patients were randomly assigned to receive triplet therapy (encorafenib, binimetinib, and cetuximab), doublet therapy (encorafenib and cetuximab), or control therapy (cetuximab with irinotecan or FOLFIRI), with the primary endpoint being the OS and ORR in the triplet-therapy group versus the control group. The median OS was 9.3 months for triplet therapy and doublet therapy, versus 5.9 months for the control group (HR for triplet vs. control = 0.60, 95% CI = 0.47 to 0.75; HR for doublet vs. control = 0.61, 95% CI = 0.48 to 0.77). The confirmed response rate was 26.8% for triplet therapy and 19.5% for doublet therapy, versus 1.8% for the control. Grade 3 or higher adverse events occurred in 65.8% for triplet therapy, 57.4% for doublet therapy, and 64.2% for the control group. Based on these results, the FDA approved the doublet regimen (encorafenib plus cetuximab) for previously treated patients with BRAF V600E–mutant mCRC [38]. The idea behind the BEACON study is based on the understanding that *BRAF* mutations can activate the *RAS/MEK/ERK* pathway, leading to *EGFR* signaling. The dual inhibition of both *BRAF* and *EGFR* prevents the activation of adaptive feedback mechanisms, enhancing the treatment efficacy. The BREAKWATER trial is currently investigating the use of encorafenib, with or without chemotherapy, versus standard care as a first-line treatment in patients with *BRAF V600E*-mutant mCRC [39]. Additionally, phase I/II clinical trials (NCT04017650 and NCT05217446) are exploring the safety and effectiveness of a treatment regimen involving encorafenib, cetuximab, and immunotherapy (nivolumab and pembrolizumab) in patients with microsatellite-stable colorectal cancer harboring the *BRAFV600E* mutation. This approach seeks to achieve a synergistic effect by combining immunotherapy to enhance the immune system’s targeting of cancer cells, encorafenib to inhibit the cancer-driving *BRAF V600E* mutation, and cetuximab to target the often overactivated EGFR pathway in colorectal cancer. The neoadjuvant study (Clinicaltrials.gov ID: NCT05710406) is a phase II/III study assessing whether combining the *RAF* inhibitor encorafenib with the *EGFR* inhibitor cetuximab improves the disease-free survival in resected, *BRAF V600E*-mutant, high-risk, stage II or III colon cancer patients post-standard adjuvant therapy. The rationale behind this study is that patients with mismatch-repair-proficient, *BRAF V600E*-mutant, high-risk, stage II or III colon cancer face a significant risk of recurrence, despite receiving standard adjuvant therapy. Prior research has shown that combining encorafenib with cetuximab improves the survival outcomes in metastatic settings. This study aims to evaluate if this combination can also enhance the disease-free survival in the earlier, resected disease setting [40].

In the *non-V600 BRAF* realm, current treatments primarily involve standard chemotherapy, but ongoing research is focusing on more effective targeted therapies. The BEAVER trial, a phase II study, is evaluating the combination of encorafenib (a BRAF inhibitor) and binimetinib (a MEK inhibitor) in advanced solid tumors, including CRC with non-V600 BRAF mutations [41]. The rationale is to block the *MAPK/ERK* pathway at multiple points for comprehensive inhibition. Finally, ASN007, an ERK1/2 inhibitor, is being tested in patients with *BRAF* fusion or *non-V600* mutations to assess the effects of direct *ERK* inhibition, a downstream effector in *BRAF*-mutated cancers [42].

### 5.2. EGFR Inhibitors

Cetuximab and panitumumab are two notable *EGFR* inhibitors. The PRIME study, a randomized phase III clinical trial with 1183 patients, tested panitumumab–FOLFOX4 versus FOLFOX4 alone in untreated wild-type (WT) *KRAS* metastatic colorectal cancer. The primary end point of the study was the PFS, and the secondary endpoint was the OS. The median PFS was significantly improved at 10 months (95% CI = 9.3–11.4) in the panitumumab–FOLFOX4 arm compared to 8.6 months (95% CI = 7.5–9.5) in the FOLFOX4 arm (HR = 0.80; 95% CI = 0.67–0.95; *p* = 0.01). There was also a trend towards better overall survival in the panitumumab group (HR = 0.83, 95% CI = 0.70–0.98; *p* = 0.03). Adding panitumumab to FOLFOX4 notably increased grade 3/4 adverse events. For WT KRAS mCRC patients, significant increases were seen in skin toxicity (37% vs. 2%), diarrhea, hypokalemia, fatigue, mucositis, and hypomagnesemia compared to the use of FOLFOX4 alone. MT *KRAS* mCRC patients showed higher skin toxicity and diarrhea in the combination group, with neutropenia being more common in patients receiving FOLFOX4 alone. Adverse events led to panitumumab discontinuation in 19% of the WT and 18% of the MT *KRAS* patients, with a few instances of grade 3 infusion reactions and no grade 4 or 5 reactions reported. Similarly, the CRYSTAL study, a phase III randomized controlled trial, evaluated the efficacy of adding cetuximab to FOLFIRI as a first-line treatment for patients with *EGFR*-expressing mCRC. The study involved 1198 patients and compared FOLFIRI plus cetuximab (*n* = 599) against FOLFIRI alone (*n* = 599). The PFS was the primary endpoint.

For patients with *RAS* wild-type tumors, the addition of cetuximab to FOLFIRI resulted in a significant improvement in the OS and PFS. The HR for the OS was 0.75 (95% CI = 0.60 to 0.93, *p* = 0.009). This was a 25% reduction in the risk of death with this combination therapy compared to FOLFIRI alone. The HR for the PFS was 0.58 (95% CI = 0.44 to 0.77, *p* < 0.001). This was a 42% reduction in the risk of disease progression. The treatment was well tolerated amongst both arms, as the safety profiles were consistent across groups, with no new safety concerns [43,44]. Cetuximab and panitumumab received FDA approval for mCRC treatment in 2004 and 2007, respectively [45].

The physical location of the tumor in the colon is also a significant prognostic indicator. Patients with left-sided mCRC respond better to *EGFR* inhibitors than those with right-sided tumors. A retrospective national study CALGB/SWOG 80405, a randomized phase III trial with 1389 patients, compared the effectiveness of cetuximab and bevacizumab in patients with wild-type *KRAS* mCRC. It found that the side of the primary tumor (right vs. left) significantly affected the OS and PFS. KRAS wild-type patients had a median OS of 34.2 months for left-sided tumors compared to 19.4 months for right-sided tumors, with an HR of 1.56 (95% CI = 1.32 to 1.84), and a PFS of 11.5 months vs. 8.9 months in left-sided tumors compared to right-sided tumors, with an HR of 1.25 (95% CI = 1.08 to 1.46). This pattern was less evident in patients with KRAS-mutant tumors, though a trend favored a better OS for left-sided tumors. The study further showed that cetuximab was more effective for left-sided, wild-type tumors, while bevacizumab showed better results for right-sided tumors in KRAS wild-type mCRC.

This illustrates the need to consider both the KRAS mutation status and the tumor location when choosing a treatment regimen [46]. The National Comprehensive Cancer Network (NCCN) thereby recommends comprehensive mutation testing in all cases of metastatic colorectal cancer to inform therapeutic strategies and enhance the prognosis. The PARADIGM study was an open-label, prospective, randomized study of 823 patients. It was designed to compare the efficacy of panitumumab or bevacizumab, combined with FOLFOX, as the first-line treatment of patients with RAS wild-type mCRC and left-sided primary tumors. The primary endpoint was the OS. It showed that *RAS* wild-type mCRC patients receiving panitumumab combined with chemotherapy (FOLFOX) had a survival advantage over those treated with a bevacizumab and chemotherapy combination in the front-line setting [(OS HR of 0.82, 95% CI = 0.68-0.99; *p* = 0.031 for panitumumab vs. bevacizumab in left-sided mCRC patients) and (OS HR of 0.84, 95% CI = 0.72–0.98; *p* = 0.030 in the full analysis set)] [47]. However, it should be noted that close to 40% of the patients who received bevacizumab in the front-line setting never received anti-*EGFR* therapy during their treatment course, which may have had an influence on the OS results. Nevertheless, patients with a left-sided, *RAS* wild-type disease should receive anti-EGFR therapy during their treatment course to benefit from this therapy option. For RAS/BRAF wild-type mCRC, a key question in CRC treatment concerns the effectiveness of bevacizumab versus an initial regimen with an EGFR inhibitor in these patients. In the first-line setting, regimens containing bevacizumab may be more effective, especially when the primary tumor is located in the right colon [48].

The KRYSTAL-1 clinical trial, a phase I/II, open-label, non-randomized clinical trial, involved patients with mCRC who harbored the *KRAS G12C* mutation and had been previously treated. The patients were allocated to receive either adagrasib monotherapy (600 mg orally twice daily) or adagrasib in combination with cetuximab. The study’s primary objective was to assess the objective response rate. The response rate in the monotherapy group was 19% (95% CI = 8 to 33), with a median response duration of 4.3 months (95% CI = 2.3 to 8.3) and a median PFS of 5.6 months (95% CI = 4.1 to 8.3). In the combination group, the response rate increased to 46% (95% CI = 28 to 66), with a median response duration of 7.6 months (95% CI = 5.7 to not estimatable) and a median PFS of 6.9 months (95% CI = 5.4 to 8.1). Grade 3 or 4 treatment-related adverse events (anemia and diarrhea) were observed in 34% of the monotherapy group and 16% of the combination group, with no grade 5 adverse events reported. This study showed that adagrasib, an inhibitor of the mutant *KRAS G12C* protein, had antitumor activity in heavily pretreated metastatic colorectal cancer patients, both as a monotherapy and in combination with cetuximab [49]. In a study comparing the sotorasib–panitumumab treatment to the standard of care in patients with mCRC with mutated *KRAS G12C*, treatment with sotorasib in combination with panitumumab significantly improved the PFS in the patients compared to the standard care. In the 960 mg sotorasib–panitumumab group, the median PFS was 5.6 months (95% CI = 4.2–6.3) with an HR for disease progression or death of 0.49 (95% CI = 0.30–0.80; *p =* 0.006) compared to the standard care. In the 240 mg sotorasib–panitumumab group, the median PFS was 3.9 months (95% CI = 3.7–5.8) with an HR of 0.58 (95% CI = 0.36–0.93; *p =* 0.03) compared to the standard care. The ORR was 26.4% (95% CI = 15.3 to 40.3) for the 960 mg sotorasib group and significantly higher than the other groups. Treatment-related adverse events of grade 3 or higher occurred in 35.8% of patients in the 960 mg sotorasib group, 30.2% in the 240 mg sotorasib group, and 43.1% in the standard care group. Skin-related toxic effects and hypomagnesemia were the most common adverse events observed in patients treated with sotorasib–panitumumab [50]. Adagrasib has a planned approval date of 21 June 2024 by the FDA [51].

There are several ongoing clinical trials targeting the *MAPK* pathway in CRC. A phase Ib/II study on avutometinib and cetuximab aims to assess the safety and efficacy of avutometinib combined with cetuximab in *KRAS*-mutated metastatic colorectal cancer patients. Avutometinib is a dual *RAF/MEK* inhibitor. The study is testing its combination with cetuximab, an *EGFR* inhibitor, in *KRAS*-mutated metastatic colorectal cancer patients.

### 5.3. HER2-Targeted Therapies

Meric-Bernstam et al.‘s study (Lancet Oncology 2019) was a phase 2a, multicenter, non-randomized, open-label, multiple-basket study of 57 patients with HER2-amplified mCRC. The patients received pertuzumab and trastuzumab. The ORR was 32% (95% CI = 20–45), with one complete response and seventeen partial responses. Grade ≥3 treatment-related adverse events occurred in 37% of the patients, with hypokalemia and abdominal pain being the most common. Besides showing that the combination of pertuzumab and trastuzumab is an effective and well-tolerated treatment in patients with heavily pretreated HER2-amplified mCRC, it pointed to the importance of molecular profiling in identifying potential therapeutic targets such as HER2 amplification in metastatic colorectal cancer [52]. This approach’s rationale is that targeting different *HER2* epitopes might impede signaling more effectively than single-agent therapy. Similarly, the HERACLES trial, which was also an open-label, phase 2 study, was conducted to evaluate the efficacy of a dual HER2 blockade with trastuzumab and with the tyrosine kinase inhibitor lapatinib in patients with KRAS exon 2 (codons 12 and 13) wild-type, HER2-positive mCRC that is refractory to standard care, including cetuximab or panitumumab. They enrolled 27 patients. This resulted in a 30% ORR (95% CI = 14–50). The median PFS was 21 weeks (95% CI = 16–32). The median OS was 46 weeks (95% CI = 33–68). Grade 3 adverse events occurred in 22% of the patients and included fatigue, a skin rash, and an increased bilirubin concentration. No grade 4 or 5 adverse events were reported [53]. The MOUNTAINEER trial, a multicenter, open-label, phase 2 study, evaluated the efficacy of tucatinib plus trastuzumab in patients with chemotherapy-refractory, HER2-positive, RAS wild-type unresectable CRC or mCRC. This study had 45 patients in cohort A, 41 in cohort B (tucatinib plus trastuzumab), and 31 in cohort C (tucatinib monotherapy), totaling 117 patients. The patients were administered tucatinib (300 mg orally twice daily) plus intravenous trastuzumab (8 mg/kg initial loading dose, then 6 mg/kg every 21 days) for cohorts A and B or tucatinib monotherapy (300 mg orally twice daily) for cohort C, with an option to add trastuzumab upon progression. This study reported an ORR of 38.1% in cohorts A and B combined (95% CI = 27.7–49.3). There were 3 complete responses and 29 partial responses. The most common adverse event was diarrhea. Grade 3 or worse adverse events were uncommon, with hypertension being the most frequent (7%). This study was based on the rationale that a selective *HER2* tyrosine kinase inhibitor could offer the targeted disruption of *HER2* signaling. The median progression-free survival was 8.2 months (95% CI = 4.2–10.3), and the median overall survival was 24.1 months (95% CI = 20.3–36.7) [54]. The MOUNTAINEER and HERACLES trials led to the FDA approval of these anti-*HER2* targeted agents for the treatment of mCRC with ERBB2 amplification.

The DESTINY-CRC01 trial, which was an open-label, phase 2 trial, assessed the efficacy and safety of trastuzumab deruxtecan (T-DXd) in patients with HER2-expressing (IHC 3+ or IHC 2+/ISH+) mCRC that progressed after at least two prior regimens. This study demonstrated the potential of antibody–drug conjugates, with trastuzumab deruxtecan achieving a 45.3% ORR (95% CI = 31.6–59.6). The median OS was 15.5 months and the duration of response was 7 months. The most common grade ≥3 adverse events were a decreased neutrophil count and anemia. Drug-related interstitial lung disease/pneumonitis occurred in eight patients (9.3%) [55]. In a phase I dose-escalation and -expansion trial designed to assess the safety and anti-tumor activity of zanidatamab, a humanized, bispecific monoclonal antibody was used in patients with various solid tumors expressing or amplifying HER2. The study concluded that HER2 is an actionable target in various cancer types, including colorectal cancer (37%; 95% CI = 27.0–48.7). No dose-limiting toxicities were observed, and the most common treatment-related adverse events were grade 1–2 diarrhea and infusion reactions [56]. The MOUNTAINEER-03 study (NCT05253651), an open-label, randomized, phase III trial, is currently examining the efficacy and safety of combining tucatinib, trastuzumab, and modified FOLFOX6 as a first-line treatment for patients with HER2-positive RAS wild-type mCRC.

## 6. Immunotherapy in CRC

Immunotherapy is particularly important in the treatment of MSI-H tumors. It is also being explored in patients with microsatellite stability (MSS). In the KEYNOTE-016 phase II trial, pembrolizumab demonstrated a significant decrease in tumor size in MSI-H colorectal cancer (CRC) as compared to MMR-proficient CRC (ORR = 33%) [57]. Similarly, the CheckMate-142 trial reported that nivolumab, with or without ipilimumab (a CTLA-4 inhibitor), led to tumor reduction in a substantial proportion of patients [58]. The KEYNOTE-177 study further solidified the use of pembrolizumab as a first-line treatment for MSI-H CRC, with an ORR of 69% (95% CI = 53 to 82). The study compared pembrolizumab with standard chemotherapy regimens and found a higher progression-free survival (PFS) rate in patients receiving pembrolizumab. The durability of the anti-PD-1 and anti-CTLA-4 response in dMMR (deficient MMR) CRC was also remarkable, with a significant number of patients not showing disease progression for over 12 months.

Beyond the MSI—high group, immunotherapy continues to be groundbreaking. However, in nonmetastatic settings, the novel combination of the neoadjuvant immunotherapy drugs botensilimab (BOT) and balstilimab (BAL) demonstrated an exceptional efficacy in treating patients with locally advanced pMMR/MSS colon and rectal cancer. Historically, these groups of patients have been less responsive to immunotherapy. This treatment regimen, which involves an Fc-enhanced anti-CTLA-4 antibody (BOT) and an anti-PD-1 antibody (BAL), led to an unusual “inside-out” (serosa-to-mucosa) pattern of tumor regression. This was characterized by the clearing of cancer cells from deeper tissue layers while confining residual tumor cells to the luminal surface. Analyses revealed significant shifts in the immune microenvironment post-treatment, indicating BOT’s role as an innate-adaptive immune activator [59].

The NICHE2 study also demonstrated the effectiveness of ipilimumab and nivolumab in neoadjuvant immunotherapy for colorectal cancers, both mismatch-repair-deficient and proficient. There were notable response rates in pMMR (30% response) and dMMR (100% response) cases [60]. In a related phase 2 study, all participants with mismatch-repair-deficient stage II or III rectal adenocarcinoma treated with dostarlimab achieved a clinical complete response (100%; 95% CI = 74 to 100), forgoing the need for further chemoradiotherapy or surgery [61].

## 7. Tumor-Agnostic-Based Targeted Therapies

### NTRK Gene Fusions

Using a pooled analysis from three phase 1/2 clinical trials, a total of 159 patients with TRK-fusion-positive cancers were treated with larotrectinib. Larotrectinib showed a 79% ORR (121 out of 153 patients) in treating *TRK*-fusion-positive solid tumors. The most common grade 3 or 4 larotrectinib-related adverse events were increased alanine aminotransferase (3% of patients), anemia (2%), and a decreased neutrophil count (2%). There were no treatment-related deaths. The study included eight patients with TRK-fusion-positive colorectal cancer. Among this CRC subset, four out of the eight (50%) patients had an objective response to larotrectinib. This is impressive, given the refractory nature of these metastatic CRC patients to conventional treatments. However, further clinical trials focused specifically on NTRK-fusion-positive CRC are needed, given the small sample size [62]. Patients with a novel *LMNA-NTRK1* rearrangement in metastatic CRC respond positively to the pan-TRK inhibitor entrectinib. Entrectinib also showed efficacy in *NTRK*-fusion-positive solid tumors in 121 adults, with a 61.2% response rate, a median DoR of 20.0 months (95% CI = 13.0–38.2), and a median PFS of 13.8 months (95% CI = 10.1–19.9). The DoR was 20% in the colorectal cancer subgroup and the PFS was 2.8 months [63]. Resistance to TRK inhibitors such as larotrectinib and entrectinib can arise due to mutations in the NTRK genes, specifically changes such as NTRK1 p. G667C and NTRK3 p. G696A, which affect key areas of the TRK proteins. Additionally, alterations in other genes involved in the MAPK pathway, such as the BRAF p. V600E mutation, the KRAS p. G12D mutation, or MET amplification, have been identified as mechanisms by which cancers can become resistant to TRK inhibition [64].

## 8. *FGFR* Alterations and *MET* Amplification

A single-arm phase II clinical trial enrolled 14 patients with FGF/FGFR-altered mCRC who had previously progressed on standard treatments. The patients received pemigatinib at a dose of 13.5 mg once daily on days 1-21 of each cycle, with an option to escalate to 18 mg in the second cycle if well tolerated. No objective responses were observed among the 12 evaluable patients in the study. The ORR was 0% (95% CI = 0–23.2), and the median progression-free survival was 9.1 weeks. Grade 3 or higher adverse events occurred in 42.9% of the patients. The most common adverse events were anemia, hyperphosphatemia, increased alkaline phosphatase, increased aspartate aminotransferase, and fatigue. While pemigatinib was found to be safe in the studied mCRC population, it did not show clinical activity. This led to the trial’s early termination [65]. While pemigatinib has shown efficacy in cholangiocarcinoma with FGFR fusion, but not in this mCRC cohort with *FGFR* alterations, there are still unanswered questions about the role of *FGFR* inhibitors in mCRC. More research is needed, potentially with more selective patient cohorts.

A phase II clinical trial (NCT03592641) is evaluating the efficacy and safety of savolitinib, a selective anti-*MET* tyrosine kinase inhibitor (TKI), in patients with *MET*-amplified metastatic colorectal cancer. The trial is targeting *RAS* wild-type mCRC patients previously treated with standard therapies, including 5-FU, oxaliplatin, irinotecan, and anti-VEGF and anti-*EGFR* antibodies.

### 8.1. Vaccines in Precision Oncology

The vaccine research in colorectal cancer is promising. The phase I AMPLIFY-201 study found that the investigational vaccine ELI-002 was effective at delaying relapses in patients with KRAS-mutated pancreatic and colorectal cancer, showing an 86% reduction in relapses (HR = 0.14, 95% CI = 0.03–0.63, *p =* 0.0167) and the death risk and a median relapse-free survival of 16.33 months. ELI-002 consists of AMP-altered mutant KRAS peptide antigens with immune-stimulating properties.

Various approaches are being investigated. Tumor cell vaccines are designed to boost the immune response and increase T-cell infiltration within tumors. DNA and mRNA vaccines are being employed to introduce tumor antigens to the host. Dendritic-cell vaccines utilize mature dendritic cells. They prime tumor-specific T cells for an effective immune response [66]. The current clinical trials exploring various innovative mechanisms include a study focused on personalized neoantigen cancer vaccines combined with immune checkpoint inhibitors, aiming to enhance molecular responses and monitor the progression-free survival (clinical trial identifier: NCT05141721). Another trial investigated a combination immunotherapy regimen that included the TriAdeno vaccine and retifanlimab to assess its safety and the objective response rate in metastatic cases (clinical trial identifier: NCT06149481). A study on the safety and efficacy of the PolyPEPI1018 vaccine combined with atezolizumab is focusing on administration safety and efficacy measures, including the response rate and survival (clinical trial identifier: NCT05243862).

### 8.2. Role of Germline Testing in CRC

Approximately 70% of CRC cases are sporadic. Distinguishing between sporadic and hereditary CRC is important due to differing management strategies, especially with hereditary CRCs following a pathway characterized by MMR gene defects that lead to MSI and a high mutation rate. The study of inherited CRC has, however, been instrumental in advancing our understanding of the genetics involved in both familial and sporadic forms of colon cancer [67]. For instance, Lynch syndrome, with a lifetime CRC risk of up to 80% in affected individuals, accounts for approximately 3% of all CRC cases [68]. The discovery of germline mutations in MMR genes as the cause of Lynch syndrome helped identify hMLH1 hypermethylation as being involved in about 15% of sporadic CRCs [69].

Another notable hereditary condition is familial adenomatous polyposis. This is attributed to mutations in the APC gene. It is responsible for less than 1% of all CRC cases, yet nearly 100% will develop CRC if left untreated. It is, therefore, important to screen and identify these genetic conditions through germline testing [70]. The current guidelines suggest that all CRC patients should be screened for microsatellite instability due to the cost-effectiveness of testing and the potential for improved outcomes. This screening is performed via immunohistochemistry (IHC) for MMR proteins or microsatellite instability testing (PCR) [71].

### 8.3. Liquid Biopsy in mCRC

A liquid biopsy holds great potential across the continuum of care in mCRC, from the initial diagnosis and molecular profiling to monitoring the response to therapy and detecting disease recurrence. A pivotal role of liquid biopsies is in guiding first-line treatment decisions in mCRC, particularly in choosing targeted therapies based on the molecular characteristics of the tumor. Circulating tumor DNA (ctDNA) can reveal mutations in genes such as KRAS, NRAS, and BRAF and help tailor personalized treatment strategies. In a phase I/II prospective multicenter study, 34 patients with metastatic CRC were tissue-tested as RAS wild-type and treated with cetuximab; a ctDNA analysis performed as part of this study revealed additional RAS mutations in three patients and *BRAF* or rare RAS mutations in six others. These mutations were previously undetected in tumor tissue and are correlated with significantly poorer outcomes: patients with *RAS/BRAF* mutations at baseline had a shorter progression-free survival (1.8 months vs. 4.9 months, *p* < 0.001) and overall survival (3.1 months vs. 9.4 months, *p* = 0.001) compared to those without these mutations [72]. Another study showed that *RAS/BRAF* mutations in ctDNA were associated with a poorer progression-free survival (PFS) in subsequent first-line therapy (hazard ratio [HR] = 3.351; 95% CI = 1.172 to 9.576). This supports the potential of ctDNA as a minimally invasive method for dynamic genotyping and making prognostic predictions in mCRC patients in clinical practice [73]. A liquid biopsy can also help in guiding treatment decisions beyond the first-line setting, such as the detection of rare mutations such as NTRK or MET and for monitoring disease evolution and resistance. For example, the detection of RAS mutations in ctDNA during treatment with an *EGFR* inhibitor can be potentially helpful in guiding treatment adjustments. Their levels in the bloodstream have been linked to less favorable survival outcomes. Their detection and quantification could thereby serve as a non-invasive method to assess disease progression. A liquid biopsy can also inform rechallenge strategies with targeted therapies, allowing clinicians to reintroduce treatments such as anti-EGFR monoclonal antibodies when resistance mutations are no longer detectable, thereby potentially extending the efficacy of these drugs [74,75]. In a multicenter phase 2 trial (NCT02296203), 28 patients with RAS and BRAF wild-type mCRC who previously responded to, and then became resistant to, an irinotecan-and-cetuximab-based regimen were retreated with cetuximab plus irinotecan as a third-line therapy. This study achieved a response rate of 21% for the rechallenge. The majority of the patients that showed at rechallenge had *RAS* and *BRAF* wild-type CRC in the ctDNA analysis. The patients with RAS wild-type ctDNA had a significantly longer PFS (median = 4.0 vs. 1.9 months; HR = 0.44; *p* = 0.03) compared to those with *RAS*-mutated ctDNA [76]. The CHRONOS, a phase II, open-label, single-arm study that aimed to assess the efficacy of anti-*EGFR* rechallenge therapy with panitumumab in *RAS and BRAF* wild-type mCRC, also demonstrated that ctDNA can effectively guide anti-EGFR rechallenge therapy with panitumumab in mCRC patients. Of the 27 enrolled patients, 8 (30%) achieved a partial response and 17 (63%) attained disease control. This was a favorable result compared to standard third-line treatments [77]. While larger trials are needed to validate this approach against standard treatments, it is evident that these studies present a compelling case for the potential of ctDNA-guided therapy and anti-*EGFR* rechallenge. The use of a liquid biopsy for MSI testing in CRC is also an exciting area of research, with the potential to enhance non-invasive cancer diagnostics and personalized treatment strategies. Studies are currently exploring the feasibility and effectiveness of this technique. For example, droplet digital PCR and amplicon-based NGS have shown potential in detecting the MSI status with a high specificity and sensitivity from cfDNA and ctDNA in blood samples. This, however, still faces challenges, including technical limitations, the need for standardization, and the requirement for further clinical validation [78].

ctDNA undoubtedly offers a more convenient method and a reduced procedural risk while overcoming sampling bias. It is particularly important for assessing spatial and temporal intra-tumoral heterogeneity with minimal invasiveness. This ability to capture both the spatial and temporal heterogeneity of tumors places a ctDNA analysis at the forefront of precision medicine for mCRC. Its integration in clinical trials is also becoming increasingly crucial for patient selection and biomarker identification. Liquid biopsies are anticipated to become a standard method for monitoring genomic alterations during tumor evolution, especially with exposure to molecular targeted drugs [79,80].

## 9. Resistance Mechanisms

While the initial responses to *BRAF* inhibitors can be promising, resistance typically develops, involving the reactivation of the *MAPK* pathway despite the blockade of *BRAF*, EGFR, and/or MEK. This resistance is attributed to the tumor’s molecular heterogeneity, often presenting multiple concurrent resistance mechanisms. The key mechanisms include the hyperactivation of alternative receptor tyrosine kinases such as *HER2* or *MET*; feedback loops leading to ERK hyperphosphorylation; structural modifications of BRAF; gene amplifications and acquired mutations, including *KRAS* and *BRAF*; and cell cycle dysregulation. Other potential contributors to resistance involve alterations in the PI3K/AKT/mTOR pathway, the overexpression of chemokine receptor 4, and the activation of the Wnt/beta-catenin pathway. Given this complexity, overcoming resistance necessitates innovative therapeutic strategies, such as combining BRAF inhibitors with agents targeting other pathways or receptors implicated in resistance; employing ERK inhibitors; and exploring novel agents such as pan-*RAF* inhibitors or *BRAF* paradox breakers [81].

Resistance to targeted anti-EGFR therapies is driven by multiple mechanisms. These include mutations in key genes such as RAS and BRAF that activate bypass signaling pathways, alterations in the EGFR receptor and its specific ligands that diminish drug binding, and the activation of alternative growth pathways such as MET and HER2, which provide cancer cells with escape routes from therapy. Cetuximab resistance from ERBB2 is due to either ERBB2 amplification or increased heregulin, which sustains ERK1/2 signaling. This undermines the effectiveness of EGFR therapies and underscores the importance of HER2 testing in RAS/BRAF wild-type mCRC patients [82]. Additionally, cellular processes such as the epithelial-to-mesenchymal transition further complicate the resistance scenario by reducing the cancer cells’ reliance on EGFR signaling. To counter this multifaceted resistance, several tailored approaches have been used, and they include combining therapies to target diverse molecular aberrations and adapting treatment plans based on the genetic profile of the tumor [83].

## 10. Conclusions

While chemotherapy remains the cornerstone of first-line treatment in mCRC, the advent of targeted therapies marks the dawn of a promising era. Personalized medicine is rapidly transitioning from concept to practice, significantly influencing oncology’s future landscape. With ongoing research into integrating targeted therapies into first-line treatments, including HER2 and BRAF inhibitors, and the emerging utility of circulating tumor DNA for real-time molecular profiling, there has been a palpable shift towards more tailored therapeutic strategies. This evolution in targeted treatments offers a promising future where cancer care is not just about treating the disease, but about targeting it with precision, science, and insight. As such, especially in a metastatic setting, every clinician should strive to check for genetic mutations in all patients due to their treatment implications and consider a longitudinal liquid-biopsy-guided management strategy.

## Figures and Tables

**Table 1:** PICO table.

Pt Population	Intervention	Comparators	Outcomes Studied
Patients with mCRC	Molecular-targeted therapies	Standard care, chemotherapy	OS, PFS, ORR, and adverse events

**Table 2:** Molecular alterations in colorectal cancer.

Molecular Alteration	Prevalence	Diagnostic/Prognostic Implications	Associated Therapies
RAS mutations	40–45%	Poor prognosis, resistance to EGFR inhibitors	Targeted therapies (e.g., anti-EGFR for RAS wild-type)
BRAF V600E	8%	Poor prognosis, associated with more aggressive disease	BRAF inhibitors (e.g., encorafenib) + MEK inhibitors (e.g., binimetinib)
KRAS G12C	3%	Specific target for newer therapies, implications for treatment choice	KRAS G12C inhibitors (e.g., sotorasib, adagrasib)
ERBB2 (HER2) amplification	2–9%	Potential for targeted therapy, resistance to anti-EGFR in some cases	HER2-targeted therapies (e.g., trastuzumab, pertuzumab)
MSI-H/d-MMR	4%	Favorable prognosis with immunotherapy, biomarker for Lynch syndrome	PD-1 inhibitors (e.g., pembrolizumab, nivolumab)
MET amplification	1–2%	Associated with resistance to EGFR inhibitors, potential target for therapy	MET inhibitors (e.g., capmatinib, crizotinib)
Non-BRAF V600E mutations	2–3%	Potential for targeted treatments	Investigational therapies, off-label use of targeted agents
ALK/ROS1/NTRK/RET fusions	0.2–2.5%	Actionable targets for fusion-directed therapies	TRK inhibitors (e.g., larotrectinib, entrectinib) for NTRK fusions, specific inhibitors for others
FGFR alterations	4%	Potential targets for therapy, associated with certain CRC subtypes with FGFR alterations	FGFR inhibitors (e.g., pemigatinib, erdafitinib)

**Table 3:** Key clinical trials in metastatic colorectal cancer.

Trial	Focus	Stats	Findings	Impact
BEACON CRC	BRAF V600E mCRC	OS: 9.3 m vs. 5.9 m; ORR: 26.8%	Triplet/doublet-therapy-improved OS and ORR	FDA approval for encorafenib + cetuximab in second-line therapy
PRIME	WT KRAS mCRC	PFS: 10 m vs. 8.6 m	Pan-FOLFOX4-enhanced PFS	First-line panitumumab use
CRYSTAL	EGFR mCRC	OS HR: 0.75; PFS HR: 0.58	Cetux-FOLFIRI-improved OS and PFS in RAS WT	Cetuximab + FOLFIRI in first-line therapy
MOUNTAINEER	HER2+ RAS WT mCRC	ORR: 38.1%	Tucatinib + trastuzumab effective in HER2+ mCRC	FDA approval in second-line therapy
MOUNTAINEER-3	HER2+ RAS WT mCRC 1st-line	Ongoing	Evaluating tucatinib + trastuzumab + chemo	Expanding first-line treatment
HERACLES	HER2+ mCRC	ORR: 30%; PFS: 21 w; OS: 46 w	Dual HER2 blockade in KRAS WT HER2+ mCRC	Further HER2 research
DESTINY-CRC01	HER2+ mCRC	ORR: 45.3%; OS: 15.5m	T-DXd effective in HER2+ mCRC	Advanced ADC use
KEYNOTE-016	MSI-H/dMMR CRC	ORR: 33%	Pembrolizumab effective in MSI-H/dMMR CRC	Pembrolizumab for MSI-H/dMMR CRC
CheckMate-142	MSI-H/dMMR CRC	Significant ORR	Nivo ± ipi effective in MSI-H/dMMR CRC	Nivo ± ipi use in MSI-H/dMMR CRC
BEAVER	Non-V60E BRAF CRC	Ongoing	Encorafenib + binimetinib efficacy	Non-V600E BRAF CRC therapies
ASN007	BRAF fusion/non-V600	Ongoing	ERK1/2 inhibitor ASN007 effects	Downstream MAPK targeting
BREAKWATER	BRAF V600E mCRC 1st-line	Ongoing	Encorafenib + cetux ± chemo vs. standard	First-line treatment options

## Abbreviations

OS	Overall Survival
PFS	Progression-Free Survival
ORR	Objective Response Rate
CRC	Colorectal Cancer
mCRC	Metastatic Colorectal Cancer
KRAS	Kirsten Rat Sarcoma Viral Oncogene Homolog
NRAS	Neuroblastoma RAS Viral Oncogene Homolog
HRAS	Harvey Rat Sarcoma Viral Oncogene Homolog
BRAF	B-Raf Proto-Oncogene
ERBB2	Erb-B2 Receptor Tyrosine Kinase 2
MSI-H	Microsatellite Instability—High
d-MMR	Deficient Mismatch Repair
NTRK	Neurotrophic Tyrosine Receptor Kinase
FGFR	Fibroblast Growth Factor Receptor
MET	MET Proto-Oncogene, Receptor Tyrosine Kinase
RET	RET Proto-Oncogene
TP53	Tumor Protein p53
APC	Adenomatous Polyposis Coli
ALK	Anaplastic Lymphoma Kinase
ROS1	c-ros Oncogene 1
PD-1	Programmed Death 1
KRAS G12C	KRAS Gly12Cys Mutation
HER2	Human Epidermal Growth Factor Receptor 2
WT	Wild-Type
HR	Hazard Ratio
m	months
nivo	Nivolumab
ipi	Ipilimumab
T-DXd	Trastuzumab Deruxtecan
ADC	Antibody–Drug Conjugate
pan	Panitumumab
cetux	Cetuximab
FOLFOX	Folinic Acid, Fluorouracil, Oxaliplatin
FOLFIRI	Folinic Acid, Fluorouracil, Irinotecan
